# An easy to construct sub-micron resolution imaging system

**DOI:** 10.1038/s41598-020-78509-6

**Published:** 2020-12-11

**Authors:** Lakhi Sharma, A. Roy, S. Panja, S. De

**Affiliations:** 1 CSIR - National Physical Laboratory, Dr. K. S. Krishnan Marg, New Delhi, 110012 India; 2 Academy of Scientific and Innovative Research (AcSIR), Ghaziabad, 201002 India; 3 Max Planck Institute for the Science of Light, Staudtstrasse 2, Erlangen, 91058 Germany; 4 Inter-University Centre for Astronomy and Astrophysics (IUCAA), Post Bag 4, Ganeshkhind, Pune, 411007 India

**Keywords:** Atom optics, Imaging techniques

## Abstract

We report an easy to construct imaging system that can resolve particles separated by $\ge$ 0.68 $\upmu$m with minimum aberrations. Its first photon collecting lens is placed at a distance of 31.6 mm giving wide optical access. The microscope has a Numerical Aperture (NA) of 0.33, which is able to collect signal over 0.36 sr. The diffraction limited objective and magnifier recollects 77% photons into the central disc of the image with a transverse spherical aberration of 0.05 mm and magnification upto 238. The system has a depth of field of 142 $\upmu$m and a field of view of 56 $\upmu$m which images a large ensemble of atoms. The imaging system gives a diffraction limited performance over visible to near-infrared wavelengths on optimization of the working distance and the distance between the objective and magnifier.

## Introduction

Studying molecular dynamics, many body physics, quantum simulation by detecting individual atoms and ions rely on high resolution, minimally aberrated optical system to form a magnified image of the object. Imaging systems are used in fluorescence microscopy and mass spectroscopy, which gained immense interest particularly for detection of biological molecules and other chemical compounds. Micron level resolution in such cases allows direct study of the molecular dynamics^1,2^. Precision spectroscopy for parity non-conservation (PNC), electric dipole moment, optical clock etc., which uses single atom^3–5^ or ion^6–10^, rely on high resolution imaging. Quantum phase transitions, quantum simulations and quantum information processing (QIP) by using atoms in an optical lattice or array of ions in an electrodynamic trap demands imaging of the individual particles.

In particular to the rapidly progressing QIP, (i) scaling up of the qubit and (ii) their individual addressing are the present challenges for which reading the individual atoms or ions is important^11^. These applications demand sub-micron resolution for detection of trapped ions^12–17^ and atoms in optical lattices^18–22^. Different approaches, such as, by measuring the current produced upon impinging of a focussed electron beam on to the sample^23^ and most commonly by setting up of a high quality imaging system are being used. In the latter case, the signal photons either from fluorescence or from absorption imaging are collected by different customized optical systems such as micro fabricated Phase Fresnel lenses (PFLs) or by using high NA diffraction limited objectives. Alt reported an objective with NA 0.29 covering 2.1% of 4$\pi$ solid angle to detect a single atom in a magneto optical trap^24^. Sortais et al. reported imaging with objective of NA 0.5 and magnification of 25^25^. Nelson et al. first reported direct observation of individual atoms in lattice sites and imaging different lattice planes using a lens of NA 0.55 and magnification 32^22^, which then became a powerful tool for such systems to study quantum dynamics. Karski et al. reported an objective with NA 0.29 and magnification of 54 to resolve atoms separated by 433 nm^26^. Using customized lenses and wavefront corrector plate, Bakr et al. achieved the highest effective NA 0.8 so far and resolution 0.6 $\upmu$m, which was pathbreaking for quantum gas microscopy^27^. For high resolution detection over large volume, Jechow et al. demonstrated use of microfabricated PFLs that obtained NA of 0.64 covering 12% of 4$\pi$ solid angle and a magnification of 615 ± 9^28^. Due to small size, PFLs can be placed close to the sample which results to higher NA and they can also be arranged in an array to extend the viewing region further^13^. Wong-Campos et al. reported the least aberrated imaging and NA 0.6 to detect ions confined in a microfabricated trap^15^.

In this article, we describe design geometry of an easy to construct imaging system using off-the-shelf optics where positions of only two lenses are critical and also obtain their important features. The design can be adapted over visible to near-infrared wavelengths in different applications which require sub-micron spatial resolution and high quality images. The described imaging system will be used to image single Ytterbium-ion using its $^{2}S_{1/2} \rightarrow ^{2}P_{1/2}$ fluorescence at the wavelength 369.5 nm in our optical clock experiment^29^.

## Design of the lens system

The wavefronts propagating with photons get deformed due to inhomogeneous refractive index of the medium. As a result, the image formed by an optical system is aberrated which can be minimized, if not cancelled^30^, by proper choices of lenses and optimization of the design parameters. For quantitative analysis, let’s consider $(y,\, z)$ and $(Y,\, Z)$ as the coordinates for exit pupil and its image, respectively, while the source is at the origin and the imaging system is along the *x* axis. Introducing polar coordinates $(\rho , \theta )$ and $(r, \phi )$ in the exit pupil and image planes, respectively, the wave aberration $W(h,\rho , \theta )$ at the exit pupil for a rotationally symmetric optical system in its image plane (*yz* plane), can be written as^31,32^:

1 $$\begin{aligned} W(h,\rho , \theta )= & {} \sum _{j,m,n}W_{klm}h^{k}\rho ^{l}\cos ^{m}\theta , \end{aligned}$$

where aperture size $\rho = \sqrt{y^{2} + z^{2}}$, $\theta$ is the azimuthal angle in the pupil plane, *h* is the image height, *j*, *k*, *l*, *m*, *n* are integers satisfying the condition $k = 2j + m$, $l = 2n + m$ and $W_{klm}$ are the aberration coefficients. Upon expanding Eq. (1), the terms associated to coefficients $W_{200}$, $W_{111}$ and $W_{020}$ represent piston, tilt and defocus, respectively, which do not contribute in case of monochromatic light source. Terms associated to $W_{040}$, $W_{131}$, $W_{222}$, $W_{220}$ and $W_{311}$ represent Seidel aberrations such as spherical, coma, astigmatism, field curvature and distortion, respectively and the remaining represents higher order distortions^31^. In case of monochromatic photons emitted by a point source located close to the imaging axis, coma and astigmatism do not play major role. Spherical aberration dominates in case of imaging fluorescence that is isotropically emitted in all directions, hence Eq. (1) simplifies to

2 $$\begin{aligned} W(\rho )= & {} \sum _{n=2}^{p}W_{02n0}\rho ^{2n}, \end{aligned}$$

where $k = 0, l = 2n, m =0$ and *p* and *n* are integers. The spatial profile of an image corresponding to a point source, given by the PSF $S(r,\,\phi ) = A(r,\,\phi )$ $A^{*}(r,\,\phi )$ is in the form of an Airy pattern for a nearly perfect optical system^33^. Here, *A* and $A^{*}$ are amplitude distribution of the image and its complex conjugate, respectively. The deformation of amplitude distribution during propagation can be obtained from pupil function as,

3 $$\begin{aligned} P(\rho , \theta )= & {} E(\rho , \theta )\,\,\,exp\big [ikW(\rho )\big ], \end{aligned}$$

where *k* is the wave vector and $E(\rho , \theta )$ is the transmittance amplitude of the optical system. Hence, the amplitude distribution is,

4 $$\begin{aligned} A(r,\, \phi )\approx & {} C\int _{0}^{2 \pi }\int _{0}^{1} P(\rho , \theta )\,\,\,exp\bigg [-i\rho \beta \cos (\theta - \phi )\bigg ]\rho d\rho d\theta , \end{aligned}$$

with $\beta$ = $\frac{\pi r}{\lambda F}$ and *C* = $\frac{-i}{4\lambda F^{2}} exp\bigg (ik\frac{r^{2}}{r_{W}}\bigg )$, where *r* = $\sqrt{Y^{2} + Z^{2}}$, $\lambda$ is photon’s wavelength, *F* is effective focal number and $r_{W}$ is radius of curvature of the wavefront at the exit pupil. Considering fully transmissive pupil i.e. $E(\rho , \theta ) = 1$ and using Eqs. (3) and (4), the PSF reduces to^31,32,34^

5 $$\begin{aligned} S(r, \lambda , F)\approx & {} \frac{\pi }{\lambda ^{2}F^{2}}\bigg |V_{0} + ikV_{1} - \frac{k^{2}}{2}V_{2}\bigg |^{2}, \end{aligned}$$

where, $V_{0} = 2\frac{J_{1}(\beta )}{\beta }$, $V_{1}= 2\int _{0}^{1}W(\rho )J_{0}(\beta \rho )\rho d\rho$ and $V_{2}= 2\int _{0}^{1}W^{2}(\rho )J_{0}(\beta \rho )\rho d\rho$. The normalized PSF reduces to

6 $$\begin{aligned} S(r, \lambda , F) = V_{0}^{2} - k^{2}(V_{0}V_{2} - V_{1}^{2}) + 0.25k^{4}V_{2}^{2}, \end{aligned}$$

which we shall use throughout this article.

The Optics Software for Layout and Optimization (OSLO) is used for design optimization of the imaging system. It is capable of performing ray tracing and estimating the mentioned quality assurance parameters of an optical assembly upon proper feeding of its components^35^. OSLO considers upto 7th order of $W(\rho )$ as given in Eq. (2) for its analysis using sequential ray tracing method^35^.

**Figure 1:**
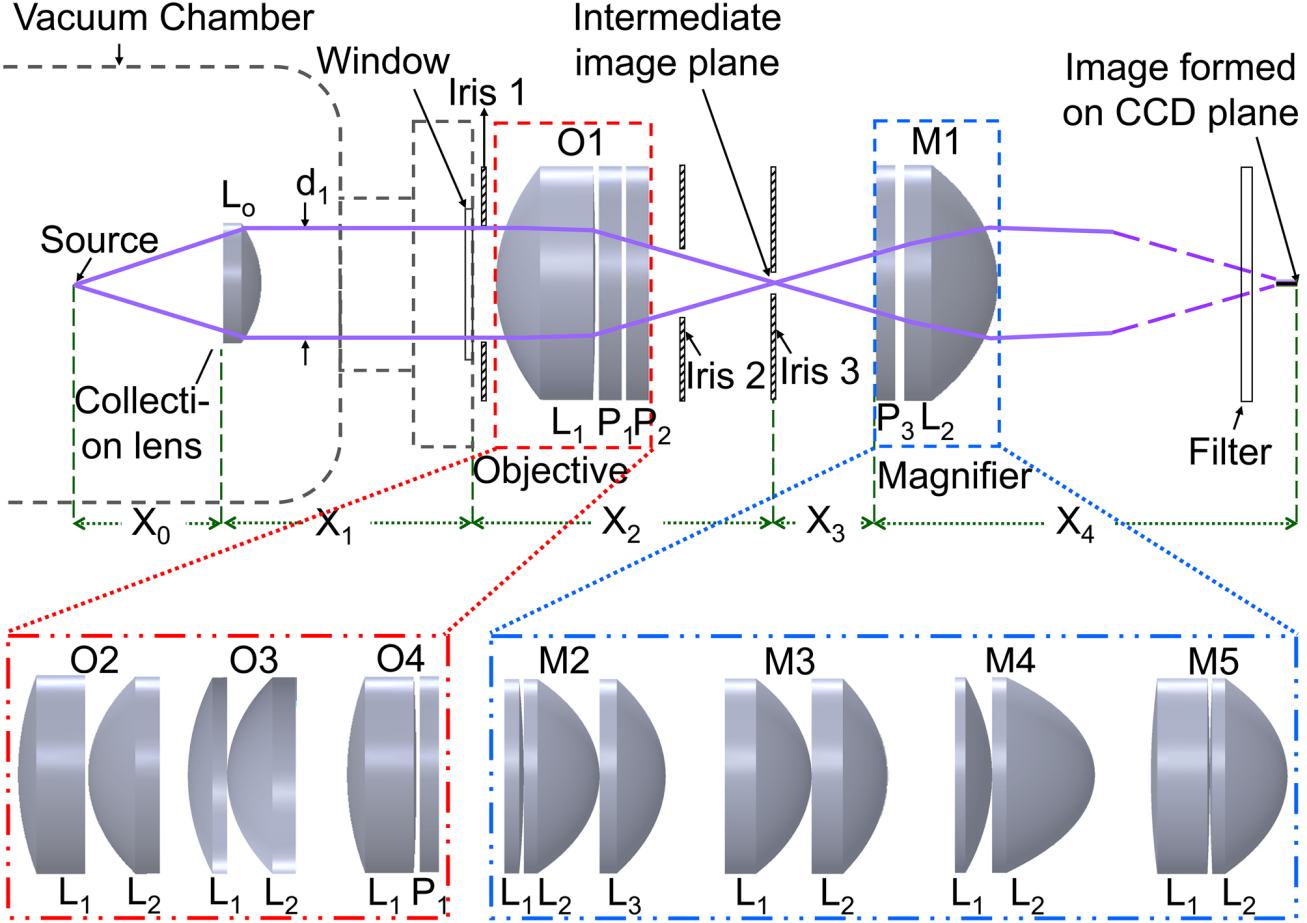
Schematic of the imaging system considering a variety of objectives O1–O4 and magnifiers M1–M5. Lenses and wavefront corrector plates (WCP) are indicated as $\hbox {L}_{i}$ and $\hbox {P}_{i}$, respectively, where *i* is their numbers. The collection lens $\hbox {L}_{\circ }$, and optics in objective, magnifier are of standard 25 mm and 50 mm diameter, respectively. For the $\hbox {L}_{\circ }$–O1–M1 combination, indicated length scales are $X_{\circ }$ = 31.6 mm, $X_{1}$ = 60.4 mm, $X_{2}$ = 58.2 mm, $X_{3}$ = 34 mm and $X_{4}$ = 4154 mm.

Optical solutions to image a single Ytterbium ion (Yb$^{+}$), as an example, which is a point like source emitting fluorescence at wavelength 369.5 nm is discussed here. The imaging system for such cases should have efficient fluorescence collection, adequate resolution $l_{R}$ and magnification *M* to distinguish individual ions and minimized aberration. Here, we consider transverse spherical aberration (SA) owing to the fact that all rays fall within the magnified image eventhough longitudinally they are not focussed at a single point. Other than SA, Strehl ratio SR and root mean square wavefront deformation $\sigma$, specify characteristics of an imaging system following^36–39^,

7 $$\begin{aligned} SR = \exp (-2\pi \sigma )^{2}, \end{aligned}$$

which can be approximated to $SR = 1 - (2\pi \sigma )^{2}$ for diffraction limited optics. For resolving two ions, diameter of central spot of the image $A_{image}$ which reduces to Airy disc diameter $A_{D}$ for a nearly perfect image should follow

8 $$\begin{aligned} A_{image} < l_{R} \times M, \end{aligned}$$

so that images do not overlap. Following the Mar$\acute{\text {e}}$chal’s Strehl approximation^40^, $SR \ge 0.8$ is acceptable, which corresponds to $\sigma \le \lambda /14$. Thus, the optics of surface roughness $\simeq$ $\lambda$/20 are recommended for constructing the imaging system. Positioning the collection lens nearer to the source enhances the fluorescence collection but that freedom is limited by available geometry which in our experiment is 31.5 mm to avoid any obstruction. In an ensemble of *N* trapped ions, minimum separation between two consecutive central ions is^41^,

9 $$\begin{aligned} l_{\circ }= & {} \bigg (\frac{e^{2}}{4\pi \epsilon _{\circ }m\omega _{s}^{2}}\bigg )^{\frac{1}{3}}\frac{2.018}{N^{0.559}}, \end{aligned}$$

where *e*, $\epsilon _{\circ }$, *m* and $\omega _{s}$ are the electron charge, free space permittivity, mass of the ion and secular frequency, respectively. The minimum separation between two species that can be resolved by a lens system is $l_{R}$ = 0.61$\lambda /NA$. In case of five ions confined in our Paul trap geometry^42,43^, as an example, $l_{\circ }$ = 1.4 $\upmu$m that demands NA $\ge$ 0.16 and as per Nyquist criterion a magnification $\ge$ 11 for a pixel size of 8 $\upmu$m $\times$ 8 $\upmu$m.

The schematic of imaging system(s) considered in our analysis is shown in Fig. 1. An aspheric lens $\hbox {L}_{\circ }$ of focal length $f_{\circ }$ = 40 mm and 25.4 mm diameter is placed inside the vacuum chamber at a working distance $X_{\circ }$ = 31.6 mm from the source. It collects 2.8% of the fluorescence, covering 0.36 sr solid angle and nearly collimates them towards the chamber window. This is followed by an objective $\hbox {O}_{i}$ outside the vacuum chamber, where *i* represents different variants, which forms an intermediate image of $A_{image}$ = 1.3 $\upmu$m at a distance $X_{2}$ from the window. The magnifier $\hbox {M}_{i}$ is placed at a spacing $X_{3}$ from the intermediate image, which forms a magnified image on the charge coupled detector (CCD) at a distance $X_{4}$ from it. After the magnifier, we use a flipper mirror to route the fluorescence either towards a Photo Multiplier Tube (PMT) or to the CCD. In both cases, it passes through an appropriate bandpass filter to transmit the desired wavelength. Distances between source and $\hbox {L}_{\circ }$; and $\hbox {O}_{i}$ to $\hbox {M}_{i}$ are critical to form the best image. An iris mounted on a YZ translation stage is placed after the viewport to obstruct the unwanted photons scattered from surface of the vacuum chamber and knife edges. Another precision iris is mounted on a three axes translation stage and placed at the intermediate image position for its spatial isolation from others. A third iris mounted on YZ translation stage is placed immediately after $\hbox {O}_{i}$ for second stage elimination of scattered photons that is useful for optical alignment of the imaging system as well.

## Results and discussions

**Figure 2:**
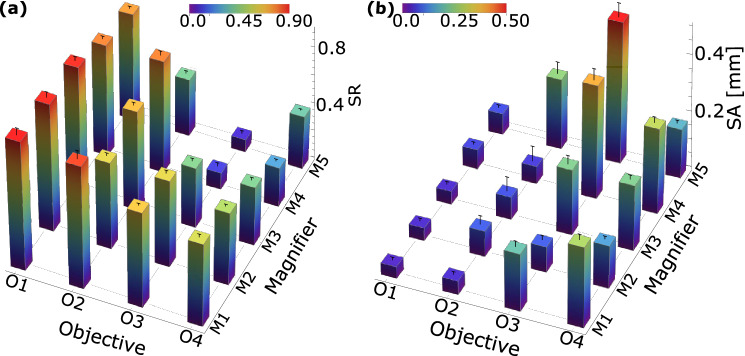
(**a**) Strehl ratio (SR) and (**b**) spherical aberration (SA) for all possible combinations of objectives (O1–O4) and magnifiers (M1–M5) those we have studied.

In this section, we discuss performances of different imaging systems that we have studied. Table 1 lists values of parameters that we obtained for different objectives and magnifiers along with simulated ion images. Throughout our analysis, the collection lens $\hbox {L}_{\circ }$ of $f_{\circ }$ = 40 mm, its position $X_{\circ }$ = 31.6 mm and $\lambda$ = 369.5 nm is fixed. To resolve the species, the imaging system should satisfy in-situ interspecies separation $l_{\circ } \gg SA$ at the intermediate image and

10 $$\begin{aligned} l_{\circ } \times M > SA, \end{aligned}$$

at the final image. Among the different objectives, O1 and O2 satisfy Mar$\acute{\text {e}}$chal’s criterion^40^ and have $\sigma$, $l_{R}$ and SA values within the acceptable range. With the available commercial lenses, O3 gives a better resolution and an acceptable SA but SR = 0.74 does not satisfy Mar$\acute{\text {e}}$chal’s criterion. O4 does not minimize SA to the acceptable limit, results to a poor SR and has a low resolving power. The resultant SR and SA for all twenty combinations between objective-magnifier pairs are shown in Fig. 2a,b, respectively. Combination of O1 with M1, M2, M3, M4 and O2 with M1, M4 results to SR > 0.8, among them O1–M1 gives the best results with SR = 0.92, $l_{R}$ = 0.68 $\upmu$m and a diffraction limited performance over M = 73 to 238; whereas O2–M1 with SR = 0.87 and $l_{R}$ = 0.7 $\upmu$m is also a probable choice but offers a comparatively lower magnification ranging from 51 to 65. For our experiment, we opt for O1–M1 combination which consists of only one aspheric lens together with two + 1$\lambda$ spherical aberration compensation plates, making the imaging system simple to construct. Using off-the-shelf SA corrector plates enhances the performance of the system, thus producing better images. The results presented in this section are corresponding to the $\hbox {L}_{\circ }$–O1–M1 system. The component and air spacing details of the system is catalogued in Table 2 (see Supplementary Fig. S1 for more details). Estimated photon transfer efficiency due to reflections from its multiple optical surfaces without any band-pass filter is 0.91 and on passing through a 370 ± 2 nm commercial bandpass filter with 25% transmissivity is 0.23.

**Table 1:** Estimated values of parameters for different lens combinations that we studied, where the magnifiers in conjunction with O1 are shown here.

Objective [*f* in mm]	$A_{image}$ ($\upmu$m)	SR	SA ($\upmu$m)	$l_{R}$ ($\upmu$m)	Image (2.5$\times$2.5) $\upmu$m$^{2}$
O1: 1 Asp [50], 2 WCP	1.3 (2)	0.93 (1)	0.7 (1)	0.68 (3)	
O2: 1 Asp [60], 1 Ach [100]	1.2 (1)	0.84 (3)	0.7 (1)	0.71 (1)	
O3: 1 Asp [100], 1 Asp [60]	1.2 (3)	0.74 (3)	0.7 (1)	0.67 (3)	
O4: 1 Ach [100], 1 WCP	1.8 (5)	0.59 (5)	2.4 (3)	1.9 (1)	

Magnifier [*f* in mm]	$A_{image}$ (mm)	SR	SA (mm)	M	Image (0.5$\times$0.5) mm$^{2}$
M1: 1 Asp [37.5], 1 WCP	0.06 (3)	0.92 (2)	0.05 (1)	110 (4)	
M2: 1 Asp [50], 1 Asp [60] 1 PC [500]	0.01 (1)	0.91 (1)	0.02 (1)	15 (1)	
M3: 1 Asp [50], 1 Asp [60]	0.04 (3)	0.89 (1)	0.04 (3)	62 (2)	
M4: 1 Asp [37.5], 1 PC [125]	0.07 (3)	0.81 (1)	0.05 (3)	77 (3)	
M5: 1 Asp [60], 1 Ach [100]	0.03 (2)	0.76 (2)	0.03 (1)	33 (1)	

*Asp* asphere, *WCP* wavefront corrector plate, *Ach* achromat, *PC* plano convex lens.

**Table 2:** Specifications of the $\hbox {L}_{\circ }$–O1–M1 lens system.

Optics	Surface no.	Radius (mm)	Spacing (mm)	Component	Material (placement)
$\hbox {L}_{\circ }$	1	$\infty$	8.0	AFL-25-40 (Asphericon)	CROWN (Vacuum)
	2	− 19.7	43.7		
Window	3	$\infty$	1.5	VPZ38SVAR-NM (Torr scientific)	Sapphire (Air)
	4	$\infty$	0		
$\hbox {L}_{1}$of O1	5	30.8	19.4	66316 (Edmund optics)	LBAL35 (Air)
	6	− 500.0	0		
$\hbox {P}_{1}$ of O1	7	$\infty$	4.0	66765 (Edmund optics)	NBK7 (Air)
	8	$\infty$	0		
$\hbox {P}_{2}$ of O1	9	$\infty$	4.0	66765 (Edmund optics)	NBK7 (Air)
	10	$\infty$	65.5		
$\hbox {P}_{3}$ of M1	11	$\infty$	4.0	66765 (Edmund optics)	NBK7 (Air)
	12	$\infty$	0		
$\hbox {L}_{2}$ of M1	13	$\infty$	19.4	69144 (Edmund optics)	LBAL35 (Air)
	14	− 29.4	4.2 $\times$ 10$^{3}$		

**Figure 3:**
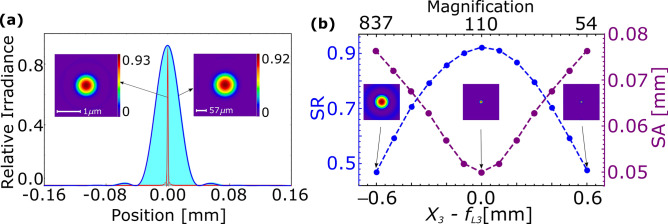
(**a**) Point spread functions (PSF) simulated by OSLO (blue), and calculated from analytical relation (filled with cyan) at the final image of the $\hbox {L}_{\circ }$–O1–M1 combination. The PSF at the intermediate image plane (red) is shown for comparison. (**b**) Variations of SR (blue) and SA (purple) due to deviation of magnifier M1 from its ideal position i.e. $X_{3} = f_{L3}$ is shown, which tunes the magnification. Images are shown in the insets for at a glance quality comparison.

Figure 3a shows PSF as simulated using OSLO and compares it to the theoretical calculation following Eq. (6) for the $\hbox {L}_{o}$–O1–M1 system, where the aberration cofficients as given in Eq. (2) are obtained from OSLO. This gives confidence to understand the imaging system and thereby helps to model a measurable PSF for a real case. PSF in the intermediate image plane is also shown, which has $A_{image}$ = 1.3 $\upmu$m. The insets show the corresponding ion images obtained by simulation. Considering Yb ion as the source, the $\hbox {L}_{\circ }$–O1–M1 system diffracts 77% of the total collected photons into the central Airy disc. Figure 3b shows tunability of M1 to vary magnification of the final image and corresponding SR and SA. Mounting the magnifier on a precision translation stage is required to tune its position at micron accuracy across its focus $f_{L3}$. Tunability of 600 $\upmu$m across $f_{L3}$ varies the magnification from 73 to 238 within the acceptable SR and SA, beyond that focussing of marginal and axial rays do not coincide, which results into greater SA and hence poorer quality, as shown in the insets of the figure.

**Figure 4:**
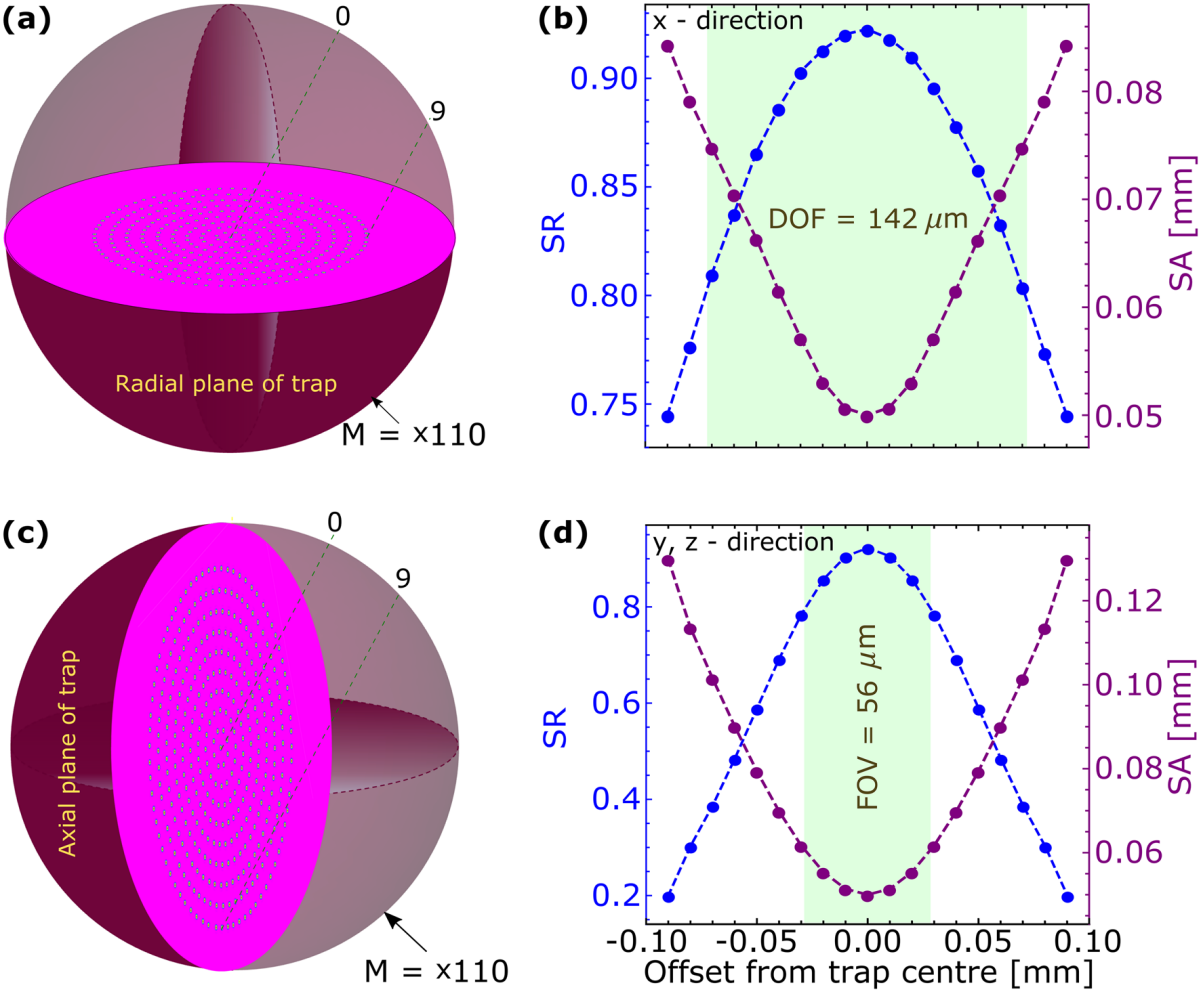
Images of trapped ions on (**a**) *xy*, (**c**) *yz* planes. (**b**) and (**d**) shows corresponding variation of SR and SA as the ion’s position deviates from best focus. Depth of field (DOF) and Field of View (FOV) are indicated.

Depth of Field (DOF) and Field of View (FOV) are the acceptable radial and axial ranges (with respect to trap’s axis i.e. z-axis), respectively, over which images with $SR \ge 0.8$ are formed^44^. These also determine number of trapped species which can be imaged at a time. In case of 19 ions trapped along a particular direction, $l_{o}$ $=$ 0.69 $\upmu$m for our trap conditions, and hence they can be resolved as $l_{R}$ = 0.68 $\upmu$m. Considering this, we show planar visualisation with simulated images of 19 ions in our trap in Fig. 4a,c for radial and axial planes, respectively. Figure 4b,d show SR and SA corresponding to these images and indicate DOF and FOV. Actual spacing between consecutive ions is considered in this analysis, which increases as they are further away from the radio-frequency nullpoint of the trap (trap centre). That together with source at out of focus results to steep change in SR and SA for the ions away from the trap centre. The $\hbox {L}_{\circ }$–O1 combination gives a DOF of 280 $\upmu$m and FOV of 94 $\upmu$m, whereas in conjunction with the magnifier these values reduce to 142 $\upmu$m and 56 $\upmu$m, respectively.

**Figure 5:**
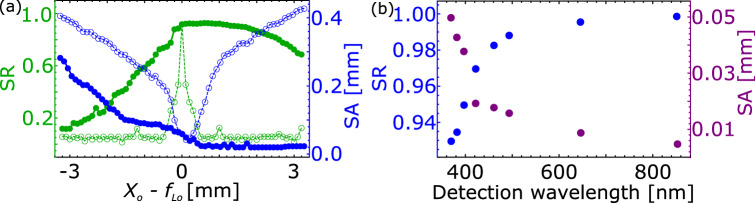
(**a**) Change in SR (green empty circle) and SA (blue empty circle) values of the images due to inaccurate positioning of the collection lens $\hbox {L}_{\circ }$ with respect to its focus $f_{L_{\circ }}$. Corresponding SR (green full circle) and SA (blue full circle) after incorporating corrections by tuning the magnifier’s position are also shown. (**b**) SR and SA at different wavelengths (corresponding to different species), e.g., 369.5 nm (Yb$^{+}$), 382 nm (Ra$^{+}$), 397 nm (Ca$^{+}$), 422 nm (Sr$^{+}$), 461 nm (Sr), 493.5 nm (Ba$^{+}$), 646 nm (Lu$^{+}$) and 852 nm (Cs).

The performance discussed is for the nominal system but the fabrication tolerance is also to be taken into account. The Peak-to-Valley (P-V) optical path difference (OPD) corresponding to an SR of 0.8 is 0.25 waves. The P-V OPD for the system, $OPD_{s}$ as obtained from the simulation is 0.19 waves; hence, to satisfy Mar$\acute{\text {e}}$chal’s criterion, the P-V OPD from fabrication tolerance, $OPD_{t}$ should be $\le$ 0.16 waves. We determined the overall fabrication tolerance of the system considering different sources and their contributions within bracket are as: radius of curvature (0.022 $\lambda$), surface irregularity (0.002 $\lambda$), element thickness/ air space (0.116 $\lambda$), refractive index (0.085 $\lambda$) and surface tilt (5 $\times$ 10$^{-5}$ $\lambda$), which results to $OPD_{t}$ = 0.14 waves. This results to an effective $OPD_{total}$ = $\sqrt{OPD_{s}^{2} + OPD_{t}^{2}}$ = 0.24 waves that corresponds to SR of 0.83 which is well within the limit. Apart from this, we have also calculated the allowable decenter and tilt tolerances for the 2 inch optics assembly to be mounted in optics tube. To maintain an SR $\ge$ 0.8, estimated tolerances in decentration and tilt are $\le$ 450 $\upmu$m and $\le$ 0.22$^{\circ }$, respectively. Deviation of $X_{\circ }$ from $f_{L_{\circ }}$ occurs from inaccurate positioning of $\hbox {L}_{\circ }$ or shifting of the trap centre. Since $\hbox {L}_{\circ }$ is inaccessible on regular basis, we studied dynamic range of its position over which SA and SR can be corrected by the external objective-magnifier combination. Figure 5a shows the change of SR and SA due to deviation of $X_{\circ }$ from $f_{L_{\circ }}$ and their post corrected values incorporated by tuning distance between O1–M1. We found, SA and SR can be significantly corrected for deviation of $X_{\circ }$ - $f_{L_{\circ }}$ from − 0.4 to 2.6 mm, which is better than the other objective-magnifier combinations and makes it user friendly. In Fig. 5b, we show that on optimization of $X_{\circ }$ and $X_{3}$, the described imaging system gives favorable results for SR and SA at different wavelengths covering from visible to near infrared corresponding to the elements Yb$^{+}$, Ra$^{+}$, Ca$^{+}$, Sr$^{+}$, Sr, Ba$^{+}$, Lu$^{+}$ and Cs. Values of other parameters describing the optical performance are stated in Table 3. Values of $\sigma < 0.07\lambda$ in all cases and a good magnification range confirms an acceptable performance of the system. Hence, the design can be adapted for applications other than Yb$^{+}$ as well.

**Table 3:** Estimated values of various optical excellence parameters obtained using OSLO for wavelengths corresponding to different atom/ion species.

$\lambda$ (nm)	Species	$\sigma$ ($\lambda$)	$l_{R}$ (µm)	$X_{\circ }$ (mm)	*M*	FOV (µm)
369.5	Yb$^{+}$	0.042 (2)	0.68 (3)	31.60 (1)	73 (1)–238 (3)	56 (2)
382	Ra$^{+}$	0.039 (2)	0.71 (1)	31.81 (1)	72 (1)–289 (3)	58 (2)
397	Ca$^{+}$	0.034 (1)	0.74 (1)	31.99 (3)	65 (1)–243 (2)	62 (2)
422	Sr$^{+}$	0.029 (3)	0.79 (1)	32.28 (3)	62 (2)–311 (1)	66 (2)
461	Sr	0.023 (1)	0.87 (1)	32.61 (1)	56 (3)–310 (1)	74 (3)
493.5	Ba$^{+}$	0.019 (2)	0.94 (3)	32.82 (2)	52 (1)–328 (5)	78 (2)
646	Lu$^{+}$	0.011 (1)	1.25 (3)	33.45 (3)	45 (3)–982 (7)	99 (3)
852	Cs	0.006 (1)	1.67 (2)	33.87 (3)	38 (4)–1192 (9)	128 (3)

## Conclusion

The design criteria together with its detailed performance of an easily buildable imaging system that can resolve particles at sub-micron level is investigated among a wide variety in this article. The finally opted lens system consists of standard catalog optics: aspheres and aberration corrector plates. This makes the system user friendly. In comparison to previous works those use multiple lenses, we achieved higher NA of 0.33 with only one asphere and two corrector plates. The system is advantageous as the image quality due to the axial misalignment of the collection lens by − 0.4 mm to + 2.6 mm can be corrected by readjustment in the later optics. The diffraction limited optics is able to recollect 77% photons to the central disc and produce images upto $\times$ 238 magnification for the objects that are separated by as minimum as 0.68 $\upmu$m with minimum spherical aberrations. Each particle in a large ensemble can be detected by this system as it has depth of field and field of view of 142 $\upmu$m and 56 $\upmu$m, respectively. In addition, the system is usable over wider wavelength range thus making it suitable to opt for different experiments.

## Supplementary information

Supplementary Information 1.

Supplementary Information 2.

## Data Availability

The data generated or analysed during this study have been included in this paper.
