# Supplementary material for: An easy to construct sub-micron resolution imaging system
Source: Sci Rep. 2020 Dec 11;10:21796. doi: 10.1038/s41598-020-78509-6 (PMC7732857; doi:10.1038/s41598-020-78509-6)
Supplement: Supplementary file 1 — Supplementary Information 1. [file 41598_2020_78509_MOESM1_ESM.pdf]

MANUSCRIPT TITLE: An easy to construct sub-micron resolution imaging system.

AUTHORS: Lakhi Sharma[1, 2], A. Roy[1, 2, 3], S. Panja[1, 2] and S. De[4*]

[1]CSIR - National Physical Laboratory, Dr. K. S. Krishnan Marg, New Delhi 110012, India

[2]Academy of Scientific and Innovative Research (AcSIR), Ghaziabad 201002, India

[3]Max Planck Institute for the Science of Light, Staudtstrasse 2, Erlangen 91058, Germany

[4]Inter-University Centre for Astronomy and Astrophysics (IUCAA), Post Bag 4, Ganeshkhind, Pune 411007, India

Supplementary information

***LENS DATA**

SRF	RADIUS	THICKNESS	APERTURE RADIUS	GLASS	NOTE
OBJ	--	31.600000	1.0000e-07	AIR	
AST	--	8.000000	12.500000 A	CROWN	
2	-19.700000	43.740000	12.500000	AIR	AFL25-40
3	--	1.500000	16.000000	SAPPHIRE	
4	--	--	16.000000	AIR	
5	30.850382	19.400000	22.500000	L-BAL35	66316
6	-500.000000	--	22.500000	AIR	
7	--	4.000000	22.500000	N-BK7	66765
8	--	--	22.500000	AIR	
9	--	4.000000	22.500000	N-BK7	66765
10	--	31.505802	22.500000	AIR	

11	--	34.000000	0.023183 S	AIR	
12	--	4.000000	22.500000	N-BK7	66765
13	--	--	22.500000	AIR	
14	--	19.400000	23.000000	L-BAL35	
15	-29.456000	37.787797	23.000000	AIR	69144
IMS	--	4.1542e+03	0.361155 S		

***SURFACE NOTES**

2	AFL25-40
5	66316
7	66765
9	66765
12	66765
15	69144

***CONIC AND POLYNOMIAL ASPHERIC DATA**

SRF	CC	AD	AE	AF	AG
2	-6.7000e-01	--	--	--	--
5	-6.2213e-01	--	--	--	--
15	-1.4314e+00	--	--	--	--

***ASPHERIC SURFACE DATA**

2 ASP ASR 16 - SYMMETRIC GENERAL ASPHERE

AS0 -- AS1 -- AS2 -1.7200e-06 AS3 -8.7400e-10

AS4 -- AS5 -- AS6 -- AS7 --

AS8 --

5 ASP ASR 16 - SYMMETRIC GENERAL ASPHERE

AS0 -- AS1 -- AS2 -- AS3 -1.3795e-10

```
     AS4   -3.1250e-13 AS5         --     AS6         --     AS7         --
     AS8         --
  8  ASP ASR   16 - SYMMETRIC GENERAL ASPHERE
     AS0         --     AS1         --     AS2   -4.4361e-09 AS3         --
     AS4         --     AS5         --     AS6         --     AS7         --
     AS8         --
 10  ASP ASR   16 - SYMMETRIC GENERAL ASPHERE
     AS0         --     AS1         --     AS2   -4.4361e-09 AS3         --
     AS4         --     AS5         --     AS6         --     AS7         --
     AS8         --
 13  ASP ASR   16 - SYMMETRIC GENERAL ASPHERE
     AS0         --     AS1         --     AS2   -4.4361e-09 AS3         --
     AS4         --     AS5         --     AS6         --     AS7         --
     AS8         --
 15  ASP ASR   16 - SYMMETRIC GENERAL ASPHERE
     AS0         --     AS1         --     AS2   -4.2897e-06 AS3
1.5537e-10
     AS4   -1.9632e-13 AS5    7.3504e-17 AS6         --     AS7         --
     AS8         --
```

***PARAXIAL CONSTANTS**

```
Effective focal length:  -36.440437
Lateral magnification:  109.532080
Numerical aperture:        0.003014
Gaussian image height:   1.0953e-05
Working F-number:        165.915318
Petzval radius:          -22.909006
Lagrange invariant:      3.4968e-08
```

***WAVEFRONT**

```
   PKVAL OPD     RMS OPD   STREHL RATIO
    0.191128    0.046076    0.919567
```
