# Supplementary material for: An easy to construct sub-micron resolution imaging system
Source: Sci Rep. 2020 Dec 11;10:21796. doi: 10.1038/s41598-020-78509-6 (PMC7732857; doi:10.1038/s41598-020-78509-6)

**MANUSCRIPT TITLE:**An easy to construct sub-micron resolution imaging system.

**AUTHORS:** Lakhi Sharma[1, 2], A. Roy[1, 2, 3], S. Panja[1, 2] and S. De[4*]

[1]CSIR - National Physical Laboratory, Dr. K. S. Krishnan Marg, New Delhi 110012, India

[2]Academy of Scientific and Innovative Research (AcSIR), Ghaziabad 201002, India

[3]Max Planck Institute for the Science of Light, Staudtstrasse 2, Erlangen 91058, Germany

[4]Inter-University Centre for Astronomy and Astrophysics (IUCAA), Post Bag 4, Ganeshkhind, Pune 411007, India

## Supplementary information

## SUPPLEMENTARY FIGURE S1:

(a) Lens data entries in Surface Data of OSLO software

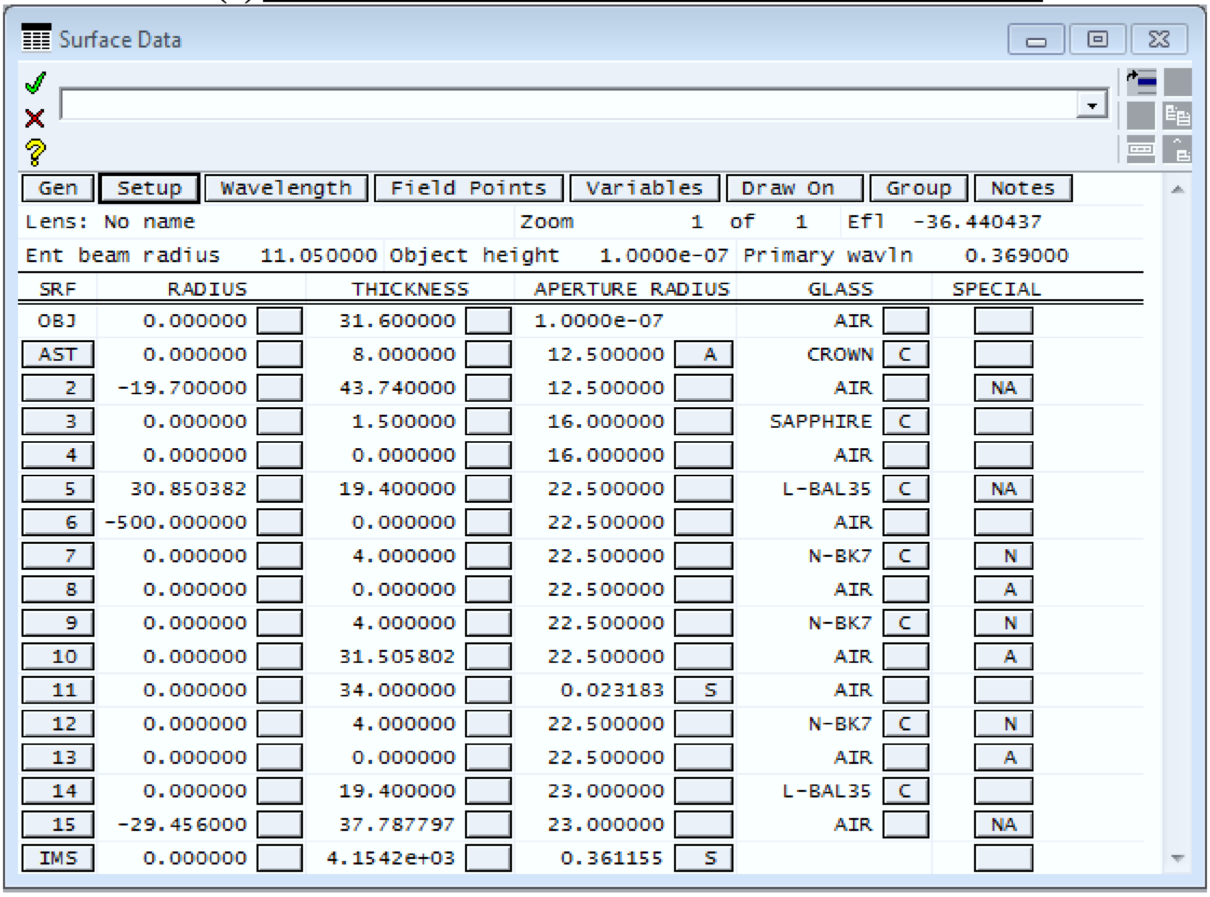


| SRF | RADIUS | THICKNESS | APERTURE RADIUS | | GLASS | | SPECIAL |
|---|---|---|---|---|---|---|---|
| OBJ | 0.000000 | 31.600000 | 1.0000e-07 | | AIR | | |
| AST | 0.000000 | 8.000000 | 12.500000 | A | CROWN | C | |
| 2 | -19.700000 | 43.740000 | 12.500000 | | AIR | | NA |
| 3 | 0.000000 | 1.500000 | 16.000000 | | SAPPHIRE | C | |
| 4 | 0.000000 | 0.000000 | 16.000000 | | AIR | | |
| 5 | 30.850382 | 19.400000 | 22.500000 | | L-BAL35 | C | NA |
| 6 | -500.000000 | 0.000000 | 22.500000 | | AIR | | |
| 7 | 0.000000 | 4.000000 | 22.500000 | | N-BK7 | C | N |
| 8 | 0.000000 | 0.000000 | 22.500000 | | AIR | | A |
| 9 | 0.000000 | 4.000000 | 22.500000 | | N-BK7 | C | N |
| 10 | 0.000000 | 31.505802 | 22.500000 | | AIR | | A |
| 11 | 0.000000 | 34.000000 | 0.023183 | S | AIR | | |
| 12 | 0.000000 | 4.000000 | 22.500000 | | N-BK7 | C | N |
| 13 | 0.000000 | 0.000000 | 22.500000 | | AIR | | A |
| 14 | 0.000000 | 19.400000 | 23.000000 | | L-BAL35 | C | |
| 15 | -29.456000 | 37.787797 | 23.000000 | | AIR | | NA |
| IMS | 0.000000 | 4.1542e+03 | 0.361155 | S | | | |

(b) Paraxial Setup Editor of OSLO Software

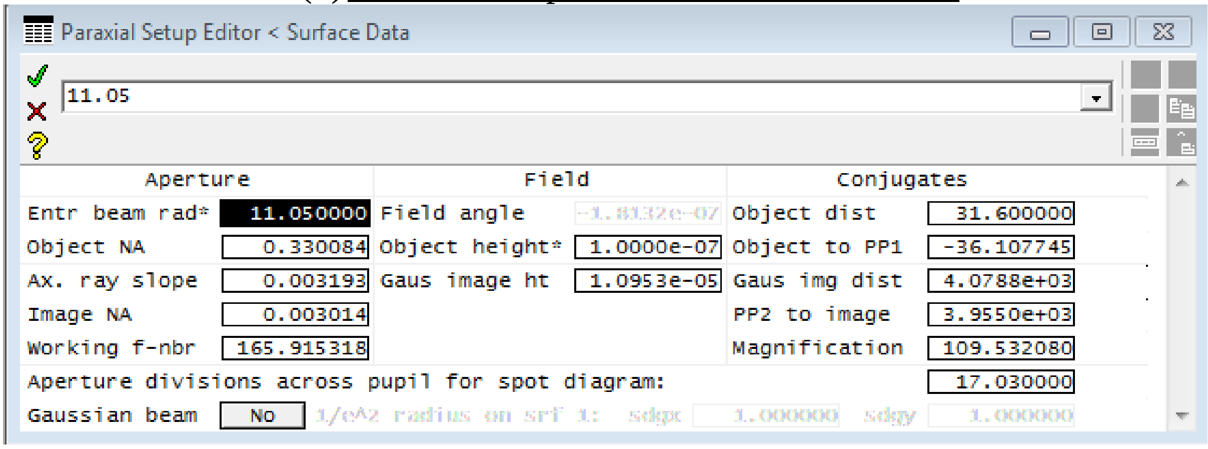

Supplement: Supplementary file 2 — Supplementary Information 2. [file 41598_2020_78509_MOESM2_ESM.pdf]
